# Knowledge of the risks, hygiene habits and perceived health issues associated with pesticide mixing and application among smallholder farmers

**DOI:** 10.1371/journal.pone.0347531

**Published:** 2026-04-30

**Authors:** Shade J. Akinsete, Oluwaseun T. Adejumo, Mumuni Adejumo, Haruna Musa Moda, Stella Ibifunmilola

**Affiliations:** 1 Department of Environmental Health Sciences, Faculty of Public Health, University of Ibadan, Nigeria; 2 Department of Environmental Health and Safety, University of Doha for Science and Technology, Qatar; 3 Department of Health Professions, Manchester Metropolitan University, United Kingdom; Universitas Airlangga, INDONESIA

## Abstract

Smallholder farmers’ exposure to pesticide can be minimized by their hygiene behaviour during pesticide application. The aim of this study was to determine knowledge of the risks, hygiene habits and perceived health issues associated with pesticide mixing and application among smallholder farmers in Nigeria. A cross-sectional study was conducted on 162 smallholder farmers from Ibarapa North Local Government Area, using a validated structured questionnaire through a two-stage sampling method. Data were analyzed using descriptive statistics and chi-square test at p = 0.05. Farmers’ mean age was 42.4 ± 12.3 years, 85.0% were male while 32.9% had tertiary education. Most (95.1%) farmers acknowledged that pesticides affect human health and 63.1% read, understood and followed pesticides label. Notably, only 4.9% acknowledged that banned or restricted pesticides cannot be used. Respondents identified pesticide exposure routes as inhalation (74.7%), dermal (15.4%), oral (1.9%) and eye contact (0.6%). Mean knowledge score was 7.9 ± 2.7 and 54.3% had poor knowledge of pesticide risk. Majority of farmers had direct skin contact with pesticide (83.3%) while 21.3% wore pesticide contaminated farm cloth home. Respondents’ use of personal protective equipment (PPE) during pesticide mixing and application were: goggles (10.1%), coverall (29.0%), head cover (22.9%) and gloves (41.8%). Additionally, unsafe disposal of empty pesticide containers on farm was common among the farmers (42.6%). Mean hygiene habit score was 5.8 ± 1.9 and 85.2% had unsafe hygiene habit. Respondents’ (90.9%) who had poor knowledge of pesticide risk was significantly (p = 0.025) higher among those who had unsafe hygiene habit during pesticide mixing and application. Reported symptoms by farmers included: dizziness > itchy eye > headache = skin irritation > nausea = coughing, during or after pesticide mixing and application. Farmers’ knowledge about pesticide risk and safe hygiene habit was poor. Farmers’ health may be at risk, hence appropriate hygiene habit and use of PPE should be strictly adhered to during pesticide mixing and application.

## 1. Introduction

Pesticides have been commonly used to promote food security among farming communities as viable means of crop protection to improve yield and quality [1–4]. However, there are concerns regarding the increased and indiscriminate use of pesticides especially in low– to middle–income countries [2,4,5] of which some of the pesticide in use are classed as highly hazardous pesticides (HHPs) hence banned or their use are highly restricted. Excessive use, poor farmers’ knowledge, improper handling and unsafe practices of pesticides among farmers in Africa is becoming increasingly disturbing [3,5,6]. Lack of knowledge and awareness among farmers in Africa have led to frequent use of banned pesticides that include HHPs based on WHO risk classification system [3,6,7]. This action among farmers is further heightened by the inadequate regulatory mechanisms and weak enforcement within the region [3]. Relatedly, farmers that are constantly exposed to pesticides may not have adequate knowledge of its occupational risk associated with lack of adherence to safety precaution when handling these chemicals. Hence the need for proactive measure aimed at regulating the use of pesticides and strengthen the monitoring of banned substance still in circulation.

Owing to the frequent use of banned and or restricted pesticides, especially organochlorines (OCPs) and organophosphates (OPPs) residues have been reported in air [8,9], water [10], soil [8], sediment [10], blood [11,12], urine [13,14] and other secondary matrices [11] in Africa. These studies are growing evidence regarding the ubiquity of pesticides in the environment and its associated health impact. Furthermore, storage of pesticides including OPPs (e.g., Chlorpyrifos) and OCPs (e.g., Endosulfan) have been reported in or around farmer’s residence in many low– to middle–countries [2,4], including Africa [14,15]. Other reported unsafe practices among farmers include the absence or inadequate use of personal protective equipment (PPE), improper disposal, and reuse of empty pesticides containers [16–19].

In addition, due to poor personal safety adherence during pesticide application, farmers are frequently exposed to pesticide residues and when either inhaled, ingested or via skin contact, such present acute or chronic effect to the human body at some point [20,21] thereby impacting on farmers overall quality of life. Several pathways have been established whereby pesticide exposure occurs among farmers, including during mixing, loading and farm application [4,6,21]. As a result of close contact, farmers and farm workers are at higher risk of exposure to pesticides [13,21,22], consequently constituting a vulnerable group whose health may be seriously compromised [21]. Despite the provision of local and international guidelines and legislation regarding safe use of pesticide, exposure among farmers and farm works especially in developing countries has become an important occupational risk partly due to poor hygiene practices. Previous studies reported poor hygiene practices and behaviors among rural farmers in southwest Nigeria [19]; farm workers in southwest Ethiopia [23]; Cocoa farmers in Ghana [24]; and horticultural farmers in Meru County, Kenya [25]. Several factors linked to farmers poor hygiene adherence during handling and application of pesticide include socio-demographic factors [26], high cost of equipment maintenance and incompatibility and or unavailability of personal protective equipment [27].

Nigeria has been ranked as the largest importer of pesticide in Africa, during the 2020 farming season, 147,446 tons of pesticides were imported into the country [28]. The informal nature of farming activity in the country, makes it difficult to ascertain the frequency of pesticide application among farmers. Despite this shortcoming, vast number of individuals are involved in farming activities in Nigeria. Although unsafe pesticide handling practices among smallholder farmers have been documented in many regions, evidence from farmers in rural communities in Nigeria remains limited. This study contributes to closing this gap by assessing farmers’ knowledge of pesticide risks, hygiene habits during mixing and application, and perceived health issues. It is imperative to comprehend these in order to establish content-specific and locally relevant interventions that speak to farmers’ life experiences—an issue that is sometimes overlooked in the international literature. Hence the study aimed to determine farmers’ knowledge regards occupational risks, hygiene habits and perceived health issues associated with pesticide mixing and application among smallholder farmers of Ibarapa North Local Government Area, in Nigeria.

## 2. Methodology

### 2.1. Study area

The study was carried out in Ayete and Tapa towns in Ibarapa North Local Government Area (INLGA), Oyo State, Nigeria. Ayete town (7° 32′ 34.296″N and 3° 13′ 21.468″E) is the headquarters of INLGA which is 72 km south west of Ibadan and 60 km north east of Abeokuta. This region is best described as undulating land scattered with hills, ridges, inselbergs and rock outcrops with pockets of low-lying plains and valleys [29]. The climate is tropical with annual rainfall ranging between 1500–2000 mm and the relative humidity is > 80% (morning) and between 50–70% (afternoon). The mean annual temperature of the area is about 27°C [29]. The INLGA is an agricultural area with farming as the main occupation of the inhabitants. The area is known for the production of cash crops, e.g., cocoa, citrus and oil palm, as well as arable crops, e.g., cassava, yam, maize, melon and various vegetables [30]. This study was conducted in November 2019 which was characterized by late rains.

### 2.2. Sampling procedure

A cross-sectional study involving a total of 162 consented farmers randomly selected from within these agrarian communities were interviewed. Interviews were conducted with participants who had engaged in farming activities and who had mixed and/or applied pesticides within three months prior to the data collecting period. Farmers that practice organic farming were excluded from the study. Consenting farmers who had not participated in pesticide mixing and/or application within the three months preceding the data collecting period were as well excluded. At the end, 173 farmers were assessed for their eligibility and 162 farmers who met the inclusion criteria were interviewed making the response rate of 93.6%.

### 2.3. Data collection instrument

A validated, interviewer administered questionnaire was used to collect information about farmers’ knowledge and safety practice regards pesticides handling and application [5,31]. The questionnaire was designed to collect information on socio-demographic characteristics, history of pesticide use, 15-point scale knowledge of pesticide risk (Scores of >7 was rated as Good), 13-point scale hygiene habit of pesticides (Scores of >6 was classified as Good), and reported illness associated with the use of pesticides. Knowledge regards occupational risks was considered based on participants awareness level of the potential hazards and health risks related to the exposure to pesticides during the mixing and application process based on the following indicators: awareness about the effect of pesticide exposure on human health, environment, and ability to read and follow pesticides instruction/ direction on the labels, and awareness that any banned or restricted pesticides cannot be used. Identification of pesticide route of exposure (e.g., inhalation, dermal, oral and eye contact) were included. Each of the item on the indicators was assigned 1-point making a total of 15-point. Knowledge score was categorized as good (scores >7) and poor (scores ≤7).

Hygiene habits assessed include farmers practices and behaviors aimed at reducing exposure to pesticide residues and contaminants during or after mixing and application. To this, 13-items were considered in the questionnaire that include habits such as eat while mixing or spraying, drinking during mixing or spraying, smoking while mixing or spraying, taking bath after mixing or spraying, washed farm cloth separately from others, skin contact with pesticide, wear farm cloth contaminated with pesticide home and personal protective equipment worn during mixing and application of pesticide. Points were assigned to each of the items, hygiene habit score was computed and rated as unsafe hygiene habit (scores ≤6) and safe hygiene habit (scores >6), respectively.

Perceived Health Issues is the subjective assessment or belief about the potential health effects associated with pesticide exposure during mixing and application and measured using questionnaire. The indicators used were the health symptoms experienced by the farmers’ during or after mixing and application of pesticides in the last 3 month prior to the survey. These symptoms were headache, dizziness, coughing, skin irritation, itchy eye, fatigue, stomachache and nausea. The questionnaire was translated into Yoruba language (and back translated to English), and pre-tested at Asejire farm settlement, Ibadan, Oyo State. Cronbach’s Alpha was used to test for the consistency of the questionnaire and the instrument was improved afterwards for its effectiveness.

### 2.4. Data collection methods

The farmers had an informal association however members neither follow any set standards of practice nor receive any specialized training about pesticide application other than learning as apprentices on the job. To establish rapport with the farmers, formal introduction and protocols were observed to reassure the association members and seek their support and permission that guarantee the study success. At the initial meeting organized, the association leaders solicited members towards the study and explained the study rationale and its associated benefit to the farmers. Two research assistants were trained on how to administer the questionnaire and ensure they both have good understanding regards interviewing skills, how to review questionnaire to ensure completeness and related ethical consideration during the administration of the research instrument prior to the start of the data collection process. For each administered questionnaire, the study objectives were explained to the participants while anonymity and confidentiality were assured and signed consents was obtained from individual farmers prior to the start of the questionnaire administration. Efforts were made to avoid respondent influencing each other regards their response choices and research assistants ensured that all the questionnaires were duly completed by respondents by crosschecking immediately.

### 2.5. Data management and analysis

Data collected was sorted, checked for completeness and accuracy, compiled, entered and analyzed using Statistical package for Social Sciences (SPSS) version 20. Categorical variables were presented using percentages while mean and standard deviation were used to present continuous variables. Chi square test was used to analyze the association between farmers’ sociodemographic characteristics, knowledge category and farmers’ hygiene habits. Ordinary logistic regression analysis was carried out to measure the influence of respondents’ educational status, smoking habit, access to training, knowledge and attitude on safe pesticide handling and application practices. The statistical significance tests were set at p = 0.05.

### 2.6. Ethical consideration

The study was conducted in accordance with all ethical procedures and the protocol was approved by the Joint Ethical Committee of University of Ibadan and University College Hospital, Ibadan, Nigeria (UI/EC//19/0434). Permission to conduct the study was obtained from the farmers’ groups in the selected communities (Tapa and Ayete) within Ibarapa North Local Government Area and informed consent was obtained from the eligible farmers. The respondents were informed of their right to withdraw from the study at any time and confidentiality of the information was maintained by using assigned codes. Additional information regarding the ethical, cultural, and scientific considerations specific to inclusivity in global research is included (see Checklist in S1 File).

## 3. Results

### 3.1. Socio-demographic characteristics of farmers and history of pesticide use

Socio-demographic characteristics of farmers and history of pesticide use are presented on Table 1. Farmers’ mean age was 42.4 ± 12.2 years and 51.2% were older than 40 years. The respondents were mostly male (84.6%) and approximately 85% were married. Nearly half (48.1%) of the farmers had secondary school education while 31.5% had tertiary education, only 7.4% identified as not having formal education. All responding farmers attest to the use of pesticides as means of farm pest control and 52.5% affirmed to pesticides application on monthly basis. Relatedly, 63.0% of the farmers reported using pesticides for ≤14 years and among these respondents, 62.3% applied pesticides more than eight times during each calendar year. Knapsack manual sprayer for pesticide application was the common method (98.8%) of pesticide application among the respondents.

**Table 1:** Socio-demographic characteristics and history of pesticide use of farmers.

Socio-demography	Frequency	Percentage	Mean± SD
**Age-group (years)**			
≤ 20	9	5.6	
21–30	15	9.3	
31–40	55	34.0	42.4 ± 12.3
≥ 41	83	51.2	
**Sex**			
Male	137	84.6	
Female	25	15.4	
**Marital status**			
Married	137	84.6	
Others*	25	15.4	
**Education Level**			
No formal education	12	7.4	
Primary	21	13.0	
Secondary	78	48.1	
Tertiary	51	31.5	
**Farmland Size (Acres)**			
< 5	95	58.6	
6–10	47	29.0	
> 10	20	12.3	
**Monthly Earning** (₦)			
< 20,000	67	41.4	
20,000–40,000	67	41.4	
> 40,000	28	17.3	
**Interval of pesticide application**			
Weekly	16	9.9	
Monthly	85	52.5	
Every 3 months	61	37.7	
**Duration of using pesticide**			
≤ 14 years	102	63.0	
>14–29 years	48	29.6	
> 29 years	12	7.4	
**Number of annual pesticide application.**			
≥8 times	101	62.3	
<8 times	61	37.7	
**Application equipment.**			
Backpack sprayer	160	98.8	
Open tractor	2	1.2	

Others*= Single, Divorced, Widowed, Separated.

### 3.2. Farmers knowledge of pesticide risk

Based on participants response regards their knowledge of pesticide, 88.9% agreed that frequent exposure to pesticides has potential to impact human health and 78.4% affirmed its associated environment impact (Table 2). Despite the level of awareness regards its health and environmental effect among the participants, 68.5% still consider pesticides as indispensable for high crop yield. Among the participants, 65.4% responded that they regularly read and follow the pesticide safety instructions. However, only 27.8% of the farmers were aware of class of pesticides that are banned or restricted for use. There were high degree of awareness regards entry route of pesticide into the human body where 74.7% confirmed inhalation as the major entry route. Dermal (15.4%), oral (1.9%) and eye contact (0.6%) were scored low. Overall mean knowledge score was 7.9 ± 2.7 and more than half of the farmers (54.3%) had poor knowledge of pesticide risk (Table 2).

**Table 2:** Knowledge of pesticide risk.

Knowledge statements	Frequency (%)	Mean±SD
Pesticides affect human health	144 (88.9)	
Pesticides usually affect the environment	127 (78.4)	
Pesticides are indispensable for high crop yield	111 (68.5)	
Farmers must read, understand and follow pesticides labels	106 (65.4)	
Some pesticides are banned or restricted for use	45 (27.8)	
Pesticides that are banned or restricted cannot be used	8 (4.9)	
**Route of exposure**		
Inhalation	121 (74.7)	
Dermal	25 (15.4)	
Oral	3 (1.9)	
Eye contact	1 (0.6)	
**Knowledge Category**		
Good	74 (45.7)	7.9 ± 2.7
Poor	8854.3)	

### 3.3. Hygiene habits during pesticide mixing and/or application

Information regards hygiene habits during handling of pesticides presented in Table 3 showed 83.3% of the respondents reported that they had skin contact with pesticide at some point and 41.3% stated that children usually assist in mixing, loading and application of pesticide. When asked how individual clean used farm cloths after pesticide application, 21.3% said they travel back home in their farm cloth contaminated with pesticide residue. Other hygiene habit during mixing and/or application of pesticides mentioned were: eating (3.8%), drinking (14.6%), and lack of spraying away from wind direction (56.3%). Also, majority of the farmers reported bathing immediately after mixing and spraying (74.7%) and washing spraying cloth separately from others (86.7%). The proportion of personal protective equipment worn by the farmers during pesticide mixing and application was considered low among the participant where only 47.5% affirmed to using nose mask (47.5%), protective boots (41.8%) and gloves (41.8%), respectively. Overall mean hygiene habit score was 5.8 ± 1.9, 85.2% of the respondents had unsafe hygiene habit during pesticide mixing and/or application (Table 3).

**Table 3:** Hygiene habits during mixing and application of pesticides.

Variables	Frequency	(%)	Mean±SD
**Hygiene Habits during mixing and application**			
Eat while mixing or spraying	6	3.8	
Drink while mixing or spraying	25	14.6	
Smoke while mixing or spraying	3	1.9	
Spray with the direction of wind	89	56.3	
Bath immediately after mixing or spraying	118	74.7	
Wash spraying cloth separately from others	137	86.7	
Spill pesticides during mixing	101	63.9	
Spill pesticides during application on farm	134	84.8	
Skin contacts with pesticide	130	83.3	
Children assist in mixing, loading and application	64	41.3	
Children assisting adequately protected	38	23.5	
Wear farm cloth contaminated with pesticide home	33	21.3	
**PPE worn during mixing and application of pesticides**			
Coveralls	45	29.0	
Protective boots	66	41.8	
Glasses and goggles	16	10.1	
Gloves	66	41.8	
Nose mask	75	47.5	
Hat/head cover	36	22.9	
**Practice Category**			
Safe hygiene practice	24	14.8	5.8 ± 1.9
Unsafe hygiene practice	138	85.2	

### 3.4. Pesticide storage and disposal practices

Table 4 present pesticides storage and disposal practices among participating farmers. From the result, pesticides storage in locked chemical store was common among the group (54.9%), open shed designated for pesticides (22.8%), living area (14.8%), open field (6.2%), in a refrigerator (0.6%), and animal house (0.6%) were other forms of storage practices mentioned. Information regard management of unused and leftover (mixed, diluted) pesticides revealed 22.8% disposed excess in the field or nearby surrounding, 38.9% said they reapply excess on already spread crops, while 21.6% said they only mix required amount to minimize excess. Concerning the handling of the expired pesticides stocks, 64.2% stated that they bought only the amount needed, 13.6% said the return to retailer while 9.9% said they dispose same in the field. Three major methods of handling empty pesticide container mentioned by farmers were to either discard on the farm (42.6%), burned on the farm (22.8%) and place in trash/dumpster (11.1%) respectively.

**Table 4:** Pesticide storage and disposal practices.

Variable	N	%
**Place of storage of pesticides**		
Open shed just for pesticide	37	22.8
In the open field	10	6.2
Locked chemical store	89	54.9
Living area	24	14.8
Refrigerator, with other items	1	0.6
Animal house	1	0.6
**Management of the unused leftover (Mixed, diluted) pesticides**		
Dispose in the field	37	22.8
Mix only needed pesticides	35	21.6
Apply on other crops	63	38.9
Missing system	28	16.2
**Management the expired pesticide stocks**		
Return to retailer	22	13.6
Hazardous waste collection sites	5	3.1
Dispose in the field	16	9.9
Buy only amount needed	104	64.2
Missing system	15	9.3
**Handling of the empty pesticide containers**		
Discard on farm	69	42.6
Place in trash or dumpster	18	11.1
Burn on farm	37	22.8
Hazardous waste collection sites	16	9.9
Bury on-farm	6	3.7
Reuse for other purposes	16	9.9

### 3.5. Reported illness experienced after Mixing and Application of Pesticides in the Last 3 months

**Fig 1:**
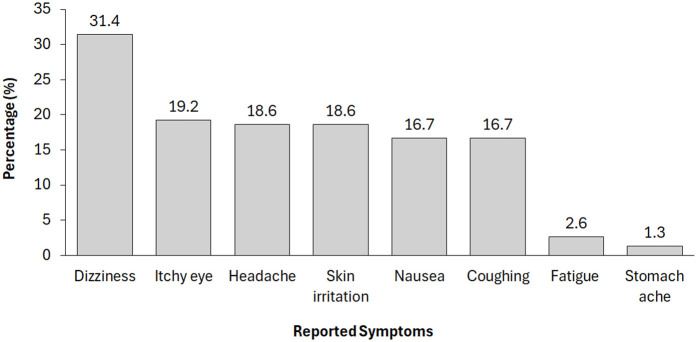
Reported symptoms experienced after mixing and application of pesticides in the last 3 months.

Fig 1 showed the reported health symptoms experienced by the farmers during or after mixing and application of pesticides in the last 3 month. The reported symptoms followed the order: dizziness > itchy eye > headache = skin irritation > nausea = coughing. Other symptoms less reported were fatigue and stomach ache.

### 3.6. Comparison between Sociodemographic characteristics, knowledge category and farmers’ hygiene habits

Safety practices score varied by farmers’ sex, educational level, farm size, types of crops grown and knowledge category (Table 5). There was no significant association between safety practices and farmers’ sex, educational level and farm size, respectively. However, proportion of farmers that grow vegetable was significantly higher among those who had unsafe hygiene habit during pesticide mixing and application (p = 0.045). Similarly, high proportion (90.9%) of farmers who had poor knowledge of pesticide risk was significantly higher among those who had unsafe hygiene habit during pesticide mixing and application (p = 0.025). Ordinary logistic regression analysis of the respondents’ types of crops grown, knowledge category and hygiene habits are presented in Table 6. The data showed that farmers who cultivate vegetables (OR = 2.195; C.I = 1.073–8.146) were more likely to practice safe hygiene habit during pesticide mixing and application than those who cultivated root crops and cereals. Also, participants who have good knowledge of pesticide risks presented better safe hygiene habit during pesticide mixing and application (OR = 2.759; C.I = 1.107–6.877).

**Table 5:** Comparison between Sociodemographic characteristics, knowledge category and farmers’ hygiene habits.

Variables	Hygiene habits		Total (%)	χ2 (Fisher’s Exact)	p-value
	Safe (%)	Unsafe %)			
**Sex**					
Male	21 (15.3)	116 (84.7)	137	0.1860	0.667
Female	3 (12.0)	22 (88.0)	25		
**Educational level**					
No Formal education	2 (16.7)	10 (83.3)	12	2.073	0.557
Primary	5 (23.8)	16 (76.2)	21		
Secondary	9 (11.5)	69 (88.5)	78		
Tertiary	8 (15.7)	43 (84.3)	51		
**Farm size (acres)**					
≤ 5	17 (19.1)	72 (80.9)	89	3.549	0.170
6-10	3 (6.8)	41 (93.2)	44		
> 10	4 (14.8)	25 (86.2)	29		
**Type of crop grown**					
Cereals	3 (27.3)	8 (72.7)	11	6.182	0.045
Root crops	6 (30.0)	14 (70.0)	20		
Vegetables	15 (11.5)	116 (88.5)	131		
**Knowledge category**					
Poor	8 (9.1)	80 (90.9)	88	5.001	0.025
Good	16 (21.6)	58 (78.4)	74		

**Table 6:** Ordinary logistic regression analysis of the respondents’ types of crop grown, knowledge category and farmers’ hygiene habits.

Types of crop grown, knowledge category	ß	Sign.	Exp(ß)	Lower Bound	Upper Bound
**Type of crop grown**					
Cereals	R.C	R.C	1.000	R.C	R.C
Root crops	0.134	0.873	1.143	0.223	5.866
Vegetables	0.929	0.038^*^	2.195	1.073	8.146
**Knowledge category**					
Poor	R.C	R.C	1.000	R.C	R.C
Good	1.051	0.029^*^	2.759	1.107	6.877

R.C = Reference Category; * Significant at 5%.

## 4. Discussion

This study determined risk, hygiene habits and perceived health issues associated with pesticide mixing and application among smallholder farmers in Nigeria. The study found that males dominated farming practice as with previous studies that reported similar findings [5,15,31–35]. This could be attributed to the task and intensive nature of farming activities hence less engaged in by females [5]. Most of the farmers in this study had formal education especially at the secondary and tertiary levels, compared with other studies that reported many farmers as illiterate or with little formal education [5,6,31]. Formal education and training are essential to enable farmers read, understand and perform some critical tasks such as calibration of sprayers and mixing of pesticides during pesticide application on the farm correctly. The high proportion of farmers that demonstrate awareness regards inhalation as a major route of exposure to pesticides is comparable to other studies in Tanzania [15] and Kuwait [31]. Fewer farmers compared to previous studies reported lack of knowledge of any route of exposure to pesticides [5,15].

Generally, the level of knowledge regards pesticide associated health risk among the sampled group in this study was generally considered as poor this further support earlier outcome reported in a previously [31]. In furtherance limited use of PPE during mixing and application of pesticides was affirmed by the participant which further corroborate other findings that reported on low use of PPE during handling and application of pesticides among farmers in other developing countries that include Tanzania [15], Ethiopia [5,6] and Bhutan [36]. Apart from cost of procuring recommended PPE, discomfort experienced during to prolong activity especially in hot and humid conditions has been identified as the main reason for farmers failure and reluctance to use PPE [5,31,37]. Poor use of PPE leads to inadequate protection during pesticide use with the implication for dermal absorption resulting from splashes and spills during mixing, loading and application pesticides [22]. Different types of PPE provide complementary levels of personal protection against dermal exposure, as such, use of multiple types of PPE for reducing exposure is required for highly toxic pesticides [22,31]. However, the protective ability of any PPE depends on proper and appropriate use. The aforementioned is imperative especially in developing countries where more toxic pesticides are used extensively [22,37].

Outcome from the study revealed that high proportion of the farmers had poor safety practices during pesticide application. This might cause environmental and human health problems. Poor pesticide handling practices has been reported to lead to harmful residues in harvested produce, soil and water contamination [38]. Our findings indicated direct exposure to pesticides by most farmers from spillage during mixing and application on farm. Furthermore, higher exposure levels will result from direct skin contact with pesticides as demonstrated in this study. Dermal exposure has been reported as one of the main routes of exposure for agricultural pesticides especially when they are readily absorbed through skin contact [22,39]. As a result, direct contact should be avoided and appropriate PPEs that reduces skin exposure should be used [39]. However, this study like previous ones demonstrate low use of coveralls during pesticide mixing or application [39]. Additional measures to reduce exposure to pesticides is bathing immediately after spraying which has been reported to reduce dermal exposure as well as pesticide metabolites in the urine [40]. Different studies have reported lower proportion of farmers observing personal hygiene after pesticide application. A high proportion of the farmers in this study bath at some point after pesticide application similar to another study [39]. On the other hand, a recent study reported a high proportion of conventional farmers who did not take a bath immediately after spraying had significantly higher pesticide parent compound residues and metabolites in their urine [40]. Furthermore, Mergia et al. [5] reported nearly all farmers (94%) in their study did not bath after mixing or spraying. Similarly, only less than half of the farmers in other studies in Ethiopia took their bath after spraying pesticides [6,35]. Not choosing to bath after spraying may be attitudinal, lack of amenity on farm site or lack of knowledge that pesticide exposure can occur via indirect skin contact from contaminated clothes.

Another unsafe practice reported among the participants is the indiscriminate disposal of empty pesticide containers on the farm, such practice could serve as secondary route of exposure as well as environmental contamination via surface runoff or leaching [5,15]. Around 10% of the farmers indicated that they reuse empty pesticide containers for other purposes. Such practices may represent a route of non-occupational exposure [15]. Generally, a high proportion (83.3%) of the farmers who reported skin contact with pesticides during mixing and application reflects poor farmer hygiene practice, causing farmers to be at increased risk of dermal exposure.

Farmers who had poor knowledge of pesticide risk was significantly higher among those who had unsafe hygiene habit during pesticide mixing and application. Conversely, farmers who have good knowledge of pesticide risks had better safe hygiene habit during pesticide mixing and application. This is an important empirical confirmation of a critical gap that could be addressed through targeted and context-specific education and training programs. Furthermore, pesticide-related health symptoms reported by respondents included dizziness, itchy eye, headache, skin irritation, nausea, coughing and others. Similar symptoms have been reported in previous studies in Kuwait [31], Ethiopia [5] and Nigeria [19]. However, a key limitation of the study is the reliance on self-reported information, which was not validated through observation or independent data sources. For example, reported use of protective clothing and self-described health issues were not verified by physical inspection or cross-checked against health center records. Consequently, the results may be affected by recall bias or social desirability bias. Future research should combine interview data with observational checks and health records to enhance accuracy.

## 5. Conclusion

Pesticides can have negative effects on the environment and human health if handled improperly, hence farmers’ knowledge of the risks, hygiene habits and perceived health issues associated with pesticide mixing and application is crucial. Although, a high proportion of participants had formal education, the study revealed poor knowledge of pesticide risks and insufficient use of personal protective equipment during mixing and application of pesticides among farmers. The implication is that education without appropriate/content-specific training on pesticide safety may not translate to proper safe practices. Moreover, our findings indicated direct exposure to pesticides occurs from spillage during mixing and application on farm. Farmers in this study displayed poor hygiene practices by improper disposal of empty pesticides containers in the field as well as high prevalence of skin contact with pesticides during mixing and application. Thus, suggesting a potentially serious public health problem in this study area. Farmers who had poor knowledge of pesticide risk did not exhibit good safety practices during pesticide application. Farmers’ may be at risk of having health challenges in the nearest future, hence the use of personal protective equipment should be strictly adhered to during pesticide handling.

## Supporting information

S1 File Checklist Inclusivity in global research questionnaire. (DOCX)

S2 File Database. (XLSX)

## References

[1] Lorenz AN, Prapamontol T, Narksen W, Srinual N, Barr DB, Riederer AM. Pilot study of pesticide knowledge, attitudes, and practices among pregnant women in northern Thailand. Int J Environ Res Public Health. 2012;9(9):3365–83. doi: 10.3390/ijerph9093365 23202693 PMC3499875

[2] Haj-Younes J, Huici O, Jørs E. Sale, storage and use of legal, illegal and obsolete pesticides in Bolivia. Cog Food Agric. 2015;1(1):1008860. doi: 10.1080/23311932.2015.1008860

[3] Sharma A, Kumar V, Shahzad B, Tanveer M, Sidhu GPS, Handa N, et al. Worldwide pesticide usage and its impacts on ecosystem. SN Appl Sci. 2019;1(11). doi: 10.1007/s42452-019-1485-1

[4] Barrón Cuenca J, Tirado N, Vikström M, Lindh CH, Stenius U, Leander K, et al. Pesticide exposure among Bolivian farmers: associations between worker protection and exposure biomarkers. J Expo Sci Environ Epidemiol. 2020;30(4):730–42. doi: 10.1038/s41370-019-0128-3 30787424 PMC8608618

[5] Mergia MT, Weldemariam ED, Eklo OM, Yimer GT. Small-scale Farmer Pesticide Knowledge and Practice and Impacts on the Environment and Human Health in Ethiopia. J Health Pollut. 2021;11(30):210607. doi: 10.5696/2156-9614-11.30.210607 34267994 PMC8276729

[6] Negatu B, Kromhout H, Mekonnen Y, Vermeulen R. Use of Chemical Pesticides in Ethiopia: A Cross-Sectional Comparative Study on Knowledge, Attitude and Practice of Farmers and Farm Workers in Three Farming Systems. Ann Occup Hyg. 2016;60(5):551–66. doi: 10.1093/annhyg/mew004 26847604

[7] Moda HM, Anang DM, Moses N, Manjo FM, Joshua VI, Christopher N, et al. Pesticide Safety Awareness among Rural Farmers in Dadinkowa, Gombe State, Nigeria. Int J Environ Res Public Health. 2022;19(13728). 10.3390/ijerph192113728 PMC9657592 36360607

[8] Degrendele C, Klánová J, Prokeš R, Příbylová P, Šenk P, Šudoma M, et al. Current use pesticides in soil and air from two agricultural sites in South Africa: Implications for environmental fate and human exposure. Sci Total Environ. 2022;807(Pt 1):150455. doi: 10.1016/j.scitotenv.2021.150455 34634720

[9] Veludo AF, Martins Figueiredo D, Degrendele C, Masinyana L, Curchod L, Kohoutek J, et al. Seasonal variations in air concentrations of 27 organochlorine pesticides (OCPs) and 25 current-use pesticides (CUPs) across three agricultural areas of South Africa. Chemosphere. 2022;289:133162. doi: 10.1016/j.chemosphere.2021.133162 34875296

[10] Ndunda EN, Madadi VO, Wandiga SO. Organochlorine pesticide residues in sediment and water from Nairobi River, Kenya: levels, distribution, and ecological risk assessment. Environ Sci Pollut Res Int. 2018;25(34):34510–8. doi: 10.1007/s11356-018-3398-8 30311117

[11] Olisah C, Okoh OO, Okoh AI. Occurrence of organochlorine pesticide residues in biological and environmental matrices in Africa: A two-decade review. Heliyon. 2020;6(3):e03518. doi: 10.1016/j.heliyon.2020.e03518 32154427 PMC7056722

[12] Afata TN, Mekonen S, Tucho GT. Evaluating the Level of Pesticides in the Blood of Small-Scale Farmers and Its Associated Risk Factors in Western Ethiopia. Environ Health Insights. 2021;15:11786302211043660. doi: 10.1177/11786302211043660 34531662 PMC8438929

[13] Motsoeneng PM, Dalvie MA. Relationship between Urinary Pesticide Residue Levels and Neurotoxic Symptoms among Women on Farms in the Western Cape, South Africa. Int J Environ Res Public Health. 2015;12(6):6281–99. doi: 10.3390/ijerph120606281 26042367 PMC4483701

[14] Wylie BJ, Ae-Ngibise KA, Boamah EA, Mujtaba M, Messerlian C, Hauser R, et al. Urinary Concentrations of Insecticide and Herbicide Metabolites among Pregnant Women in Rural Ghana: A Pilot Study. Int J Environ Res Public Health. 2017;14(4):354. doi: 10.3390/ijerph14040354 28353657 PMC5409555

[15] Lekei EE, Ngowi AV, London L. Farmers’ knowledge, practices and injuries associated with pesticide exposure in rural farming villages in Tanzania. BMC Public Health. 2014;14:389. doi: 10.1186/1471-2458-14-389 24754959 PMC3999359

[16] Alshalati, Lana M.H.D Jamal. Limited Knowledge and Unsafe Practices in Usage of Pesticides and The Associated Toxicity Symptoms among Farmers in Tullo and Finchawa Rural Kebeles, Hawassa City, Sidama Regional State, Southern Ethiopia. In Emerging Contaminants, Aurel Nuro, Ed.; IntechOpen. 2021.

[17] Nwadike C, Joshua VI, Doka PJS, Ajaj R, Abubakar Hashidu U, Gwary-Moda S, et al. Occupational Safety Knowledge, Attitude, and Practice among Farmers in Northern Nigeria during Pesticide Application—A Case Study. Sustainability. 2021;13(18):10107. doi: 10.3390/su131810107

[18] Dias AC, Silva LS, Cardoso SA, Pinheiro TMM. Knowledge and risk perception of rural workers exposed to pesticides in Teixeiras/MG: a cross-sectional study. Rev Méd Minas Gerais. 2023;33(e-33105).

[19] Oshingbade OS, Moda HM, Akinsete SJ, Adejumo M, Hassan N. Determinants of Safe Pesticide Handling and Application Among Rural Farmers. Int J Environ Res Public Health. 2025;22(2):211. doi: 10.3390/ijerph22020211 40003437 PMC11855716

[20] Tamaro CM, Smith MN, Workman T, Griffith WC, Thompson B, Faustman EM. Characterization of organophosphate pesticides in urine and home environment dust in an agricultural community. Biomarkers. 2018;23(2):174–87. doi: 10.1080/1354750X.2017.1395080 29047308 PMC6141016

[21] Hughes D, Thongkum W, Tudpor K, Turnbull N, Yukalang N, Sychareun V, et al. Pesticides use and health impacts on farmers in Thailand, Vietnam, and Lao PDR: Protocol for a survey of knowledge, behaviours and blood acetylcholinesterase concentrations. PLoS ONE. 2021;16:e0258134. 10.1371/journal.pone.0258134 PMC8483351 34591945

[22] Damalas CA, Koutroubas SD. Farmers’ Exposure to Pesticides: Toxicity Types and Ways of Prevention. Toxics. 2016;4(1):1. doi: 10.3390/toxics4010001 29051407 PMC5606636

[23] Gesesew HA, Woldemichael K, Massa D, Mwanri L. Farmers Knowledge, Attitudes, Practices and Health Problems Associated with Pesticide Use in Rural Irrigation Villages, Southwest Ethiopia. PLoS One. 2016;11(9):e0162527. doi: 10.1371/journal.pone.0162527 27622668 PMC5021266

[24] Boateng KO, Dankyi E, Amponsah IK, Awudzi GK, Amponsah E, Darko G. Knowledge, perception, and pesticide application practices among smallholder cocoa farmers in four Ghanaian cocoa-growing regions. Toxicol Rep. 2022;10:46–55. doi: 10.1016/j.toxrep.2022.12.008 36583134 PMC9792701

[25] Marete GM, Lalah JO, Mputhia J, Wekesa VW. Pesticide usage practices as sources of occupational exposure and health impacts on horticultural farmers in Meru County, Kenya. Heliyon. 2021;7(2):e06118. doi: 10.1016/j.heliyon.2021.e06118 33659728 PMC7892894

[26] Al Zadjali S, Morse S, Chenoweth J, Deadman M. Personal safety issues related to the use of pesticides in agricultural production in the Al-Batinah region of Northern Oman. Sci Total Environ. 2015;502:457–61. doi: 10.1016/j.scitotenv.2014.09.044 25282255

[27] Bhandari G, Atreya K, Yang X, Fan L, Geissen V. Factors affecting pesticide safety behaviour: The perceptions of Nepalese farmers and retailers. Sci Total Environ. 2018;631–632:1560–71. doi: 10.1016/j.scitotenv.2018.03.144 29727980

[28] Heinrich Böll Stiftung. Pesticide Atlas. Facts and figures about toxic chemicals in Agriculture. 2022 [cited 16 March 2025]. Available: https://ng.boell.org/sites/default/files/2023-03/pesticideatlas2022_nigeria_compressed.pdf

[29] Wahab B, Ogundele O. The contributions of fadama-il project to the socio-economic and infrastructural development of rural communities in ibarapa north local government area. Ibadan Plann J. 2011;1(2):165–85.

[30] Adeniyi AB, Daud AS, Amao O, Omotayo AO. Determinants of rural women’s livelihood in Ibarapa North local government area of Oyo State, Nigeria. J Hum Ecol. 2016;56:84–90.

[31] Jallow MFA, Awadh DG, Albaho MS, Devi VY, Thomas BM. Pesticide Knowledge and Safety Practices among Farm Workers in Kuwait: Results of a Survey. Int J Environ Res Public Health. 2017;14(4):340. doi: 10.3390/ijerph14040340 28338612 PMC5409541

[32] Banjo AD, Aina SA, Rije OI. Farmers’ Knowledge and Perception towards Herbicides and Pesticides Usage in Fadama Area of Okun-Owa, Ogun State of Nigeria. AJBAS. 2010;2:188–94.

[33] Adelani AO, Olajide-Taiwo FB, Adeoye IB, Olajide-Taiwo LO. Analysis of production constraints facing Fadama vegetable farmers in Oyo State, Nigeria. WJAS. 2011;7:189–92.

[34] Adesuyi AA, Njoku KL, Akinola MO, Nnodu VC. Pesticides related knowledge, attitude and safety practices among small-scale vegetable farmers in lagoon wetlands, Lagos, Nigeria. J Agric Environ Int Dev. 2018;112:81–99.

[35] Alebachew F, Azage M, Kassie GG, Chanie M. Pesticide use safety practices and associated factors among farmers in Fogera district wetland areas, south Gondar zone, Northwest Ethiopia. PLoS One. 2023;18(1):e0280185. doi: 10.1371/journal.pone.0280185 36626384 PMC9831305

[36] Monger A, Mahat K, Dorjee, Om N, Mongar P, Dorji T, et al. Assessment of exposure to pesticides and the knowledge, attitude and practice among farmers of western Bhutan. PLoS One. 2023;18(5):e0286348. doi: 10.1371/journal.pone.0286348 37252928 PMC10228793

[37] Endalew M, Gebrehiwot M, Dessie AP. Pesticide use knowledge, attitude, practices and practices associated factors among floriculture workers in Bahirdar city, North West, Ethiopia. Environ Health Insights. 2022;16:1–10. 10.1177/11786302221076250 PMC8832573 35153486

[38] Atreya K, Johnsen FH, Sitaula BK. Health and environmental costs of pesticide use in vegetable farming in Nepal. Environ Dev Sustain. 2011;14(4):477–93. doi: 10.1007/s10668-011-9334-4

[39] Liem JF, Mansyur M, Soemarko DS, Kekalih A, Subekti I, Suyatna FD, et al. Cumulative exposure characteristics of vegetable farmers exposed to Chlorpyrifos in Central Java - Indonesia; a cross-sectional study. BMC Public Health. 2021;21(1):1066. doi: 10.1186/s12889-021-11161-5 34090393 PMC8178818

[40] Tremongkoltip A, Pengpumkiat S, Kongtip P, Nankongnab N, Siri S, Woskie S. Urinary Cypermethrin Metabolites among Conventional and Organic Farmers in Thailand. Toxics. 2023;11(6):507. doi: 10.3390/toxics11060507 37368607 PMC10305172

